# Tolerance, Variability and Pharmacokinetics of Albumin-Bound Paclitaxel in Chinese Breast Cancer Patients

**DOI:** 10.3389/fphar.2018.01372

**Published:** 2018-11-29

**Authors:** Qingmei Li, Hong Zhang, Xiaoxue Zhu, Chengjiao Liu, Min Wu, Cuiyun Li, Xiaojiao Li, Lei Gao, Yanhua Ding

**Affiliations:** ^1^The First Hospital of Jilin University, Changchun, China; ^2^Phase I Clinical Research Center, The First Hospital of Jilin University, Changchun, China

**Keywords:** cancer, albumin-bound, paclitaxel, bioequivalence, variability

## Abstract

**Objective:** The aim of this study was to explore the tolerance, variability, and pharmacokinetics (PK) of albumin-bound paclitaxel (QL, HR, ZDTQ) among Chinese breast cancer patients.

**Methods:** Three randomized, open-label, two-period crossover bioequivalence studies were conducted with albumin-bound paclitaxel. Each subject received a single dose of 260 mg/m^2^ albumin-bound paclitaxel [sponsor 1 (QL, light food), sponsor 2 (HR, fasting), sponsor 3 (ZDTQ, light food); test] or Abraxane® (reference) and was monitored for 72 h. Serum concentrations of total paclitaxel and unbound paclitaxel were measured using liquid chromatography/mass spectrometry (LC/MS), and appropriate pharmacokinetic parameters were determined by non-compartmental methods. Safety assessments included adverse events, hematology and biochemistry tests.

**Results:** The bioequivalence analyses of the QL, HR, and ZDTQ products included 24, 23, and 24 patients, respectively. The mean t_1/2_ was 20.61–27.31 h for total paclitaxel. Food intake did not affect the pharmacokinetics of paclitaxel. From the comparison of total paclitaxel and unbound paclitaxel, the 90% confidence intervals (CIs) for the ratios of C_max_, AUC_0−t_, and AUC_0−∞_ were within 80.00–125.00%. The intra-subject variability ranged from 6.4–11% to 9.85–15.87% for total paclitaxel and unbound paclitaxel, respectively. Almost all subjects in the test and Abraxane® (reference) groups experienced mild or moderate adverse events. No fatal AEs or study drug injection site reactions related to these drugs were observed.

**Conclusion:** Albumin-bound paclitaxel (QL, HR or ZDTQ; test products) showed bioequivalence to Abraxane® (reference) with lower intra-subject variability, which was less than 16% in all cases, and was well-tolerated in Chinese breast cancer patients. Twenty-two patients are enough for an albumin-bound paclitaxel bioequivalence study.

## Introduction

World over, breast cancer is the most common type of malignancy among women and the second most frequent cause of cancer-related death in women^1^ (Dörfel et al., 2018; Locatelli et al., 2018). The standard therapy for patients with early breast cancer includes surgery, radiotherapy and adjuvant systemic therapy, such as anti-microtubule agents and aromatase inhibitors (Dörfel et al., 2018). However, as breast cancer is a highly heterogeneous condition, the selection of adjuvant systemic therapy depends on stage, histology and on molecular subtypes of the tumor (Dörfel et al., 2018; Locatelli et al., 2018). Current adjuvant systemic therapy options include chemotherapy, endocrine therapy for hormone receptor (HR)-positive tumors, and targeted biological agents such as trastuzumab for human epidermal growth factor receptor (HER2)-positive tumors (Dörfel et al., 2018).

Paclitaxel is an anti-microtubule agent that inhibits cell division by promoting the assembly and stabilization of microtubules (Slingerland et al., 2013). It is active against a broad spectrum of malignancies, such as non-small cell lung cancer and breast cancer (Slingerland et al., 2013; Blair and Deeks, 2015). As paclitaxel is extremely insoluble; it is solubilized in polyethoxylated castor oil for injectable preparation. However, serious and even fatal episodes of hypersensitivity with an incidence of approximately 20% have been reported with this oil (Donehower et al., 1987; Singla et al., 2002; Joerger, 2012). The introduction of premedication with corticosteroids, diphenhydramine, and H_2_ antagonists, has fortunately reduced this incidence to 2–4% (Alves et al., 2018). Still, such infusion-related hypersensitivity reactions remain a serious matter. Moreover, solubilization in polyethoxylated castor oil enhances the nonlinear pharmacokinetic (PK) activity of increasing doses of paclitaxel (Sparreboom et al., 1999; Joerger, 2012; Slingerland et al., 2013). Along with, the unbound paclitaxel is also associated with clinical toxicities, such as myelosuppression and peripheral neuropathy (Blair and Deeks, 2015).

Different modifications of drug formulations, e.g., liposomal and albumin-bound, have been studied for their ability to improve delivery of therapeutic doses, drug stability, and drug safety (Du et al., 2018). Albumin-bound paclitaxel contains protein-bound particles of paclitaxel for injectable suspension, which, by avoiding the use of polyethoxylated castor oil also eliminates the need for corticosteroid pretreatment^2^. Additionally, an improved safety profile with albumin-bound paclitaxel may facilitate the administration of higher doses^2^ (Du et al., 2018).

With the end of the patent protection period for an innovator's product, generic preparations are introduced into the market repeatedly. Thus, it has become necessary to establish the bioequivalence (BE) between two drug products with the same active moiety. Usually, determination of BE relies on comparisons of the rate and extent of absorption of a product under study (test, T) with those of an innovator's product (reference, R) (Karalis et al., 2012).

The study drugs are paclitaxel albumin protein-bound particles available as an injectable suspension (FDA, 2015). However, the production process is different, such as albumin packaging of paclitaxel process by different sponsors. The US FDA Draft Guidance on paclitaxel recommends estimation of serum unbound and total paclitaxel for BE evaluation (FDA, 2015).

As the intra-subject variability for paclitaxel among the Chinese population is unknown, we first analyzed the PK characteristics of unbound paclitaxel and total paclitaxel in Chinese breast cancer patients. Second, the present study compared the BE (rate and extent of absorption and elimination) of two 260 mg/m^2^ albumin-bound paclitaxel (test and reference) formulations as provided by the two study sponsors. Third, we analyzed the effects of sample size and intra-subject variability on the BE of unbound and total paclitaxel. Lastly, the tolerability profiles of different formulations of albumin-bound paclitaxel were assessed.

## Materials and methods

### Patients and study design

Three prospective BE studies with albumin-bound paclitaxel (injectable suspension) were conducted, with drugs provided by three different sponsors, i.e., QL (*n* = 25), HR (*n* = 25), or ZDTQ (*n* = 24), and the reference product, Abraxane®. These single-center, randomized two-period crossover, BE studies, were performed between March 2016 and March 2018, according to the US FDA guidance draft on Paclitaxel. The tolerability and PK of the test products (albumin-bound Paclitaxel, QL, HR, and ZDTQ) and Abraxane® (reference) were compared in patients with breast cancer in these three studies respectively. The inclusion criteria were: (1) age > 18 years; (2) histologic diagnosis of advanced breast cancer for which there is no curative therapy and treatment with single-agent paclitaxel has been considered appropriate by the treating physician; (3) Eastern Cooperative Oncology Group (ECOG) performance status of 0/1; (4) life expectancy of >12 weeks; and (5) complete recovery from acute toxicities of prior treatment. Subjects were excluded if they did not have adequate hematologic, kidney, and liver function (hemoglobin ≥ 90g/L [not having blood transfusion within 14 days], absolute neutrophil count ≥1.5 × 10^9^/L, blood platelet count ≥100 × 10^9^/L, total bilirubin < 1 upper limit normal [ULN], alanine aminotransferase [ALT], and aspartate aminotransferase [AST] < 2.5 × ULN [if liver metastasis, then ALT and AST ≤ 5 × ULN], creatinine ≤ 1.5 × ULN), or had received radiotherapy, chemotherapy, immunotherapy, or endocrine therapy within 4 weeks prior to the use of the study drug and residual effects were still present.

This study was carried out in accordance with the recommendations of the Good Clinical Practice and the Declaration of Helsinki. The protocol was approved by the Ethics Committee of the First Hospital of Jilin University, Changchun, Jilin, China. All subjects gave written informed consent in accordance with the Declaration of Helsinki.

**Figure 1:**
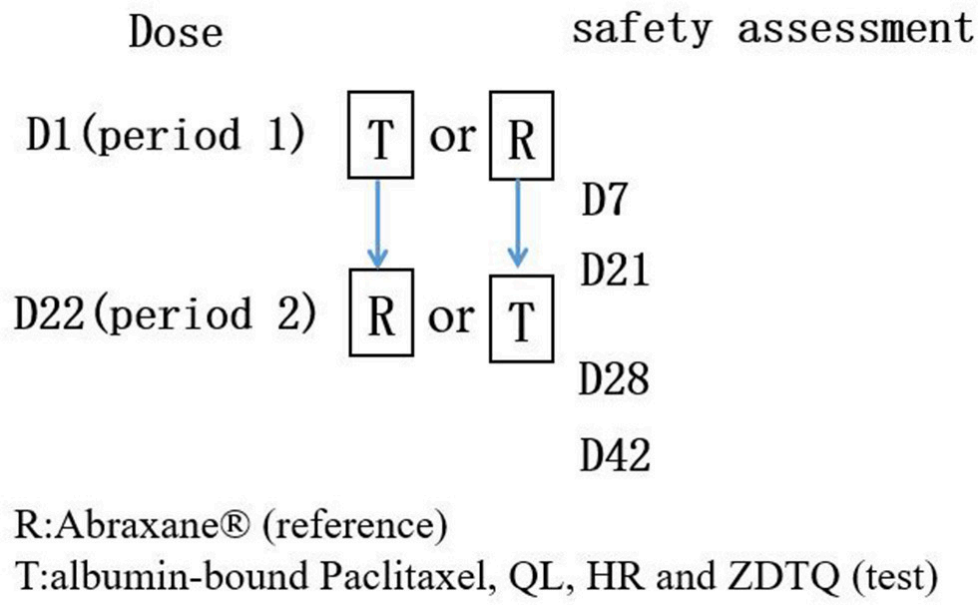
Flow chart of the BE studies. Each subject will had test product (QL, HR, or ZDTQ) and reference product (Abraxane®) at each study.

**Figure 2:**
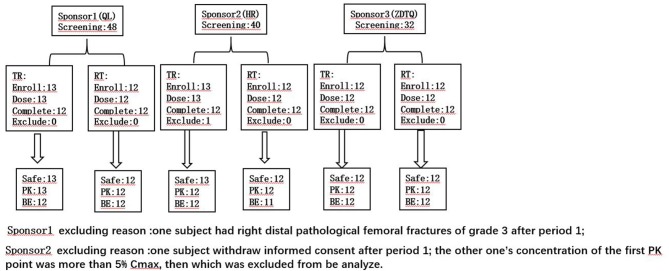
The subject number of the each analysis set. TR, D1 dosing T formulation and D22 dosing R formulation; RT, D1 dosing R formulation and D22 dosing T formulation.

A screening visit was scheduled within 14 days prior to administration of the study drug. Then the eligible subjects were admitted to the clinical research unit 1 day before dosing. Following an overnight fast of at least 8-h, subjects were randomized to receive a single intravenous dose of 260 mg/m^2^ (infusion 30 ± 3 min) of albumin-bound paclitaxel (test product; QL, HR, or ZDTQ) or Abraxane® (reference product, from the US market) in a 1:1 ratio according to a computer-generated randomization schedule for each study in the first period (Figures 1, 2). Then the same dosing method for the reference or test formulation was followed in second period, or vice versa. Each drug had a unique batch number. The washout period was of 3 weeks. Subjects were administered the drug at the same time on first day of first period and day 22 of second period (Figure 1). Albumin-bound paclitaxel by sponsor 1 (QL) and sponsor 3 (ZDTQ) was administered after breakfast (light food), whereas the HR product (sponsor 2) was administered after 8 h of fasting. Patients were carefully monitored, particularly during the infusion. Subjects were discharged after 72 h of drug administration. Blood samples for the primary PK analysis were collected prior to treatment and at specified time points during the 72-h follow-up. Subjects were followed up for safety assessment at 7 ± 1 and 21 ± 1 days.

### Estimation of sample size

According to the current US FDA guidelines, to achieve an 80–90% power (1-β) in BE studies at the 5% nominal level (α = 5%), the geometric mean ratio (GMR) is usually set to be 95–105% (Karalis et al., 2012). The coefficient of variation (CV) is evaluated as the intra-subject variability (intra-cv). The intra-cv for paclitaxel is assumed to be 20–21.3%^2^. According to the initial estimation by R software, sample size should be of 21 patients. Based on the above sample size estimation result and considering a loss to follow-up of 10% and the opinion of sponsor and investigator, the final needed sample size was considered to be 24 (Table 1) (Zhang et al., 2018a,b).

**Table 1:** Sample size estimation of these studies.

**Sponsor**	**Predicted value of bioavailability**	**α**	**1-β**	**Intra-subject variability**	**Sample size of estimation**	**Sample size in the study**
Sponsor 1 (QL)	0.95–1.05	0.05	0.8	20.00%	21	24
Sponsor 2 (HR)	0.95–1.05	0.05	0.8	21.30%	21	24
Sponsor 3 (ZDTQ)	0.95–1.05	0.05	0.8	20.00%	21	24

### Pharmacokinetic analysis

Blood samples (5 ml each time) for PK evaluation were collected into heparin anticoagulant-containing tubes within 0.2 h of initiation of albumin-bound paclitaxel infusion (pre-dose), 0.25 h after the start of infusion, immediately after stopping the infusion, and 0.75, 1, 1.5, 2, 4, 6, 8, 12, 24, 48, and 72 h after the start of infusion. Blood samples were centrifuged at 3,000 rpm for approximately 10 min at 2–8°C in a refrigerated centrifuge. The serum was stored at −70°C until analysis. The serum concentrations of total paclitaxel and unbound paclitaxel were analyzed using a validated, sensitive and specific liquid chromatography tandem mass spectrometry (LC-MS/MS) method at the Shanghai Drug Metabolism Research Center for QL and HR, and Covance for ZDTQ.

The lower limits of quantification (LLOQ) for un-bound paclitaxel and total paclitaxel were 0.2 ng/mL and 5.0 ng/mL, respectively, and the upper limits of quantification (ULOQ) for unbound paclitaxel and total paclitaxel were 2,000 ng/mL and 15,000 ng/mL, respectively for QL and HR.

The LLOQ for un-bound paclitaxel and total paclitaxel were 2 ng/mL and 10.0 ng/mL, respectively, and the ULOQ for unbound paclitaxel and total paclitaxel were 2,000 ng/mL and 10,000 ng/mL, respectively for ZDTQ. The validated concentration ranges were between the LLOQ and ULOQ.

The LC-MS/MS method and rapid equilibrium method have been validated for the determination of paclitaxel concentration in human raw serum and equilibrium serum samples (Ronghao and Jun, 2015). In each analysis batch, the quantity of quality control samples accounted for more than 5% of the unknown samples in the analysis batch. In all quality control samples, the ratio of relative deviation within 15% is more than at least 67% of all quality control samples, and the ratio of relative deviation within 15% is more than at least 50% of each concentration of quality control samples. All analysis batch quality control met the above criteria (Supplement Table 1).

Total levels of free and protein-bound paclitaxel were later quantified. Paclitaxel-D5 served as the internal standard. The analytical column was a Eclipse Plus C18 column (100 × 4.6 mm, 3.5 μm, Agilent), and the formula used for calculation of unbound paclitaxel was as follows:

$$\begin{array}{l} {\text{C}}_{\text{unbound Paclitaxel}}=\left({\text{C}}_{\text{receiver unbound Paclitaxel}}/{\text{C}}_{\text{donor Paclitaxel}}\right)\\ \times {\text{C}}_{\text{total Paclitaxel}} \end{array}$$

The inter-run assay accuracy, expressed as percent relative error for quality control samples [BIAS(%)]. The assay precision, expressed as the inter-run CV of the measured concentrations of quality control samples [relative standard deviation, RSD(%)].

All analysis batch quality control met the above criteria (Supplement table 1).

### Tolerability and safety assessments

Medical history, physical examination, electrocardiography (ECG), and laboratory test (hematology, biochemistry, and urinalysis, etc.) results were obtained at the time of screening (2–14 days before the first dose of study drug) and at 7 ± 1 and 21 ± 1 days for all subjects in each test period. Vital signs were recorded immediately before the first dose and after administration of the drug. Adverse events were recorded daily from the day of administration of the first dose through the end of the study. The National Cancer Institute Common Toxicity Criteria for Adverse Events version 4.03 was used to describe and grade all toxicities and adverse events (AEs).

### Statistical analysis

The serum concentrations vs. time data were analyzed with non-compartmental methods using WinNonlin Professional, Version 6.4 (Pharsight Corporation, NC, USA). The PK analysis used actual sample collection times. Samples below the LLOQ were set to zero before T_max_ and not detectable after T_max_ for the PK analysis. The PK parameters for paclitaxel included C_max_, area under the curve (AUC)_0−t_, AUC_0−∞_, T_max_, and T_1/2_. Descriptive statistics were calculated for PK parameters, demographics, and safety variables and these were analyzed by *t*-test or analysis of variance (ANOVA). ANOVA also was used to compare the AUC and C_max_, with factors fitted for the effect of sequence, subject within sequence, period, and treatment. The comparisons are presented in terms of the geometric least square means and the 90% confidence interval (CI). BE was established if the 90% CI of the treatment ratio was within the equivalence range of 0.8–1.25. T_max_ and T_1/2_ were analyzed with a Wilcoxon rank test. All statistical tests were performed using SAS 9.1 Statistical Package, and *P* < 0.05 was considered statistically significant.

## Results

### Subject screening, recruitment, and compliance

A total of 120 Patients with breast cancer were initially screened for these studies. Of these, 74 patients with breast cancer (*n* = 25, 25, 24) were enrolled and received the assigned study drug from sponsor 1-3 (HR, QL, ZDTQ) respectively; these patients also constituted the safety analysis set for each sponsor and 71 patients constituted the BE analysis set (Figure 2). The demographics and baseline characteristics of patients treated by the three sponsors were comparable (Table 2). Most of the study subjects were Han Chinese. The mean age of the study subjects was 48.8–52 years old.

**Table 2:** Demographic characteristics of the subjects.

**Sponsor**	**N**	**Gender (male/ female)**	**Age [years, mean (SD)]**	**Ethnicity (han/ other)**	**Body surface area [m^2^, mean (SD)]**	**Body weight[kg, mean (SD)]**	**ECOG score 0/1**
Sponsor 1(QL)	25	1/24	51.4 (8.16)	23/2	1.63 (0.15)	63.52 (9.504)	3/22
Sponsor 2 (HR)	25	2/23	48.8 (9.17)	24/1	1.58 (0.16)	60.0 (10.1)	3/22
Sponsor 3 (ZDTQ)	24	1/23	52 (7.47)	23/1	1.68 (0.16)	60.28 (10.58)	2/22
*P*		>0.05	>0.05	>0.05	>0.05	>0.05	>0.05

*ECOG, Eastern Cooperative Oncology Group*.

### Albumin-bound paclitaxel serum concentration–time profiles

**Figure 3:**
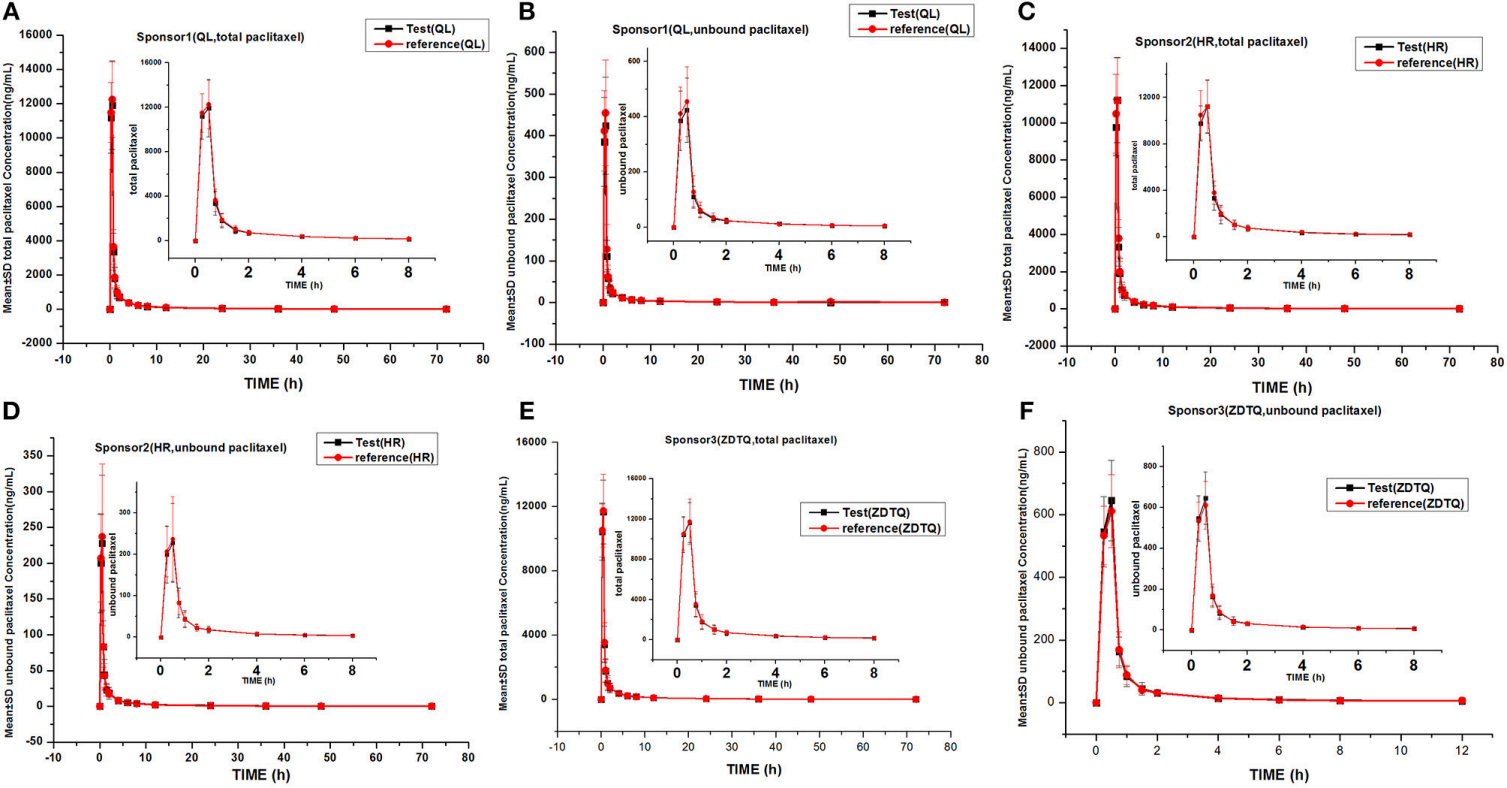
Mean serum concentration–time profiles in the study. **(A)** Sponsor 1 (QL) total paclitaxel; **(B)** Sponsor 1 (QL) unbound paclitaxel; **(C)** Sponsor 1 (HR) total paclitaxel; **(D)** Sponsor 1 (HR) unbound paclitaxel; **(E)** Sponsor 3 (ZDTQ) total paclitaxel; **(F)** Sponsor 3 (ZDTQ) unbound paclitaxel.

**Figure 4:**
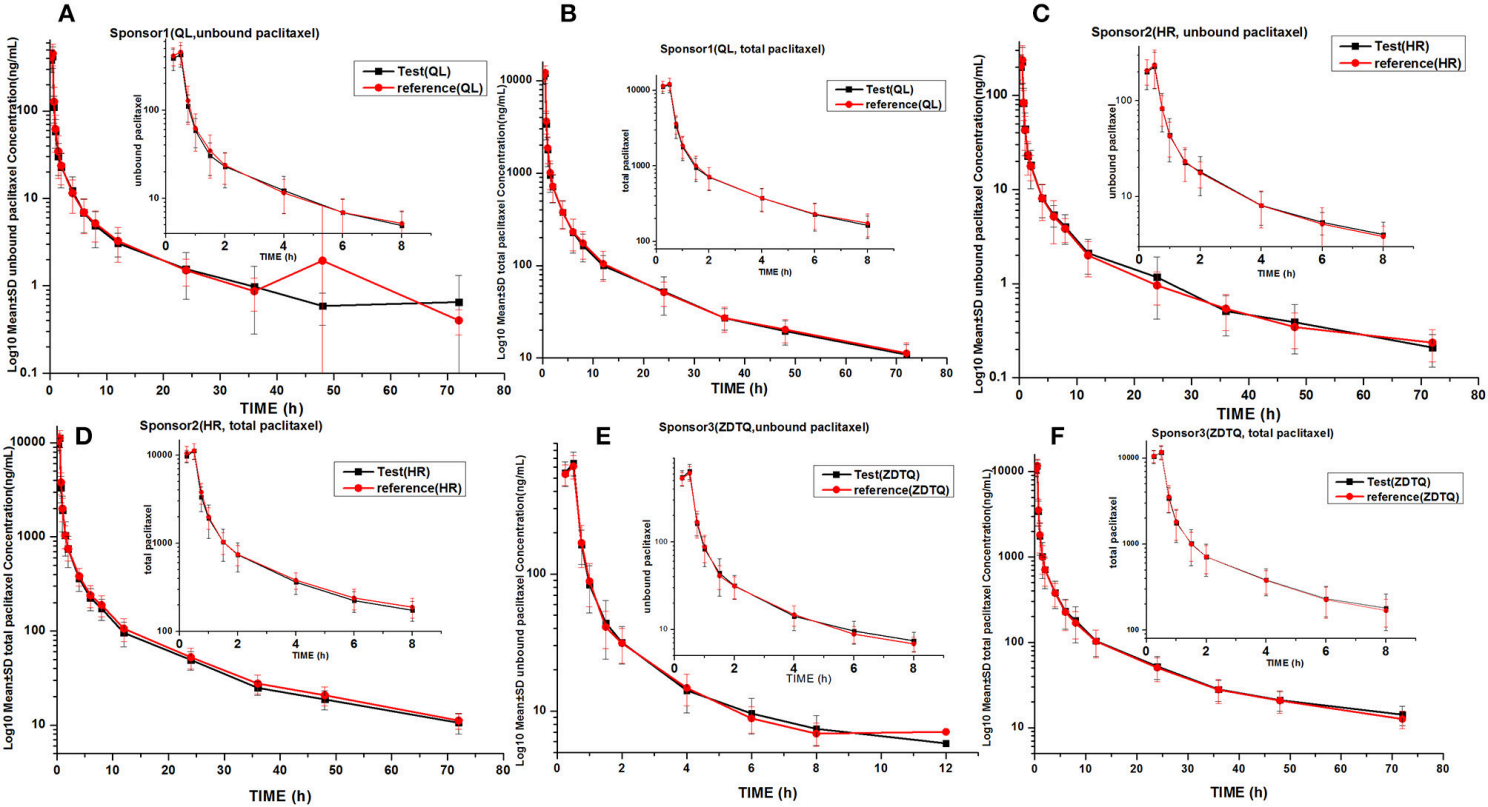
LOG10 Mean serum concentration–time profiles in the study. **(A)** Sponsor 1 (QL) total paclitaxel; **(B)** Sponsor 1 (QL) unbound paclitaxel; **(C)** Sponsor 1 (HR) total paclitaxel; **(D)** Sponsor 1 (HR) unbound paclitaxel; **(E)** Sponsor 3 (ZDTQ) total paclitaxel; **(F)** Sponsor 3 (ZDTQ) unbound paclitaxel.

The total paclitaxel and unbound paclitaxel serum concentrations increased rapidly in all study subjects and reached C_max_ at 0.5 h after the start of infusion. The serum concentrations showed a decline in a biphasic manner, which initially decreased rapidly after the end of infusion and then demonstrated a slight decrease until the lower limit of quantification. The study drugs exhibited a similar mean serum concentration–time profile in the R and T formulations in studies 1, 2, and 3 (Figures 3, 4).

### Pharmacokinetic parameters of albumin-bound paclitaxel in studies 1–3

**Figure 5:**
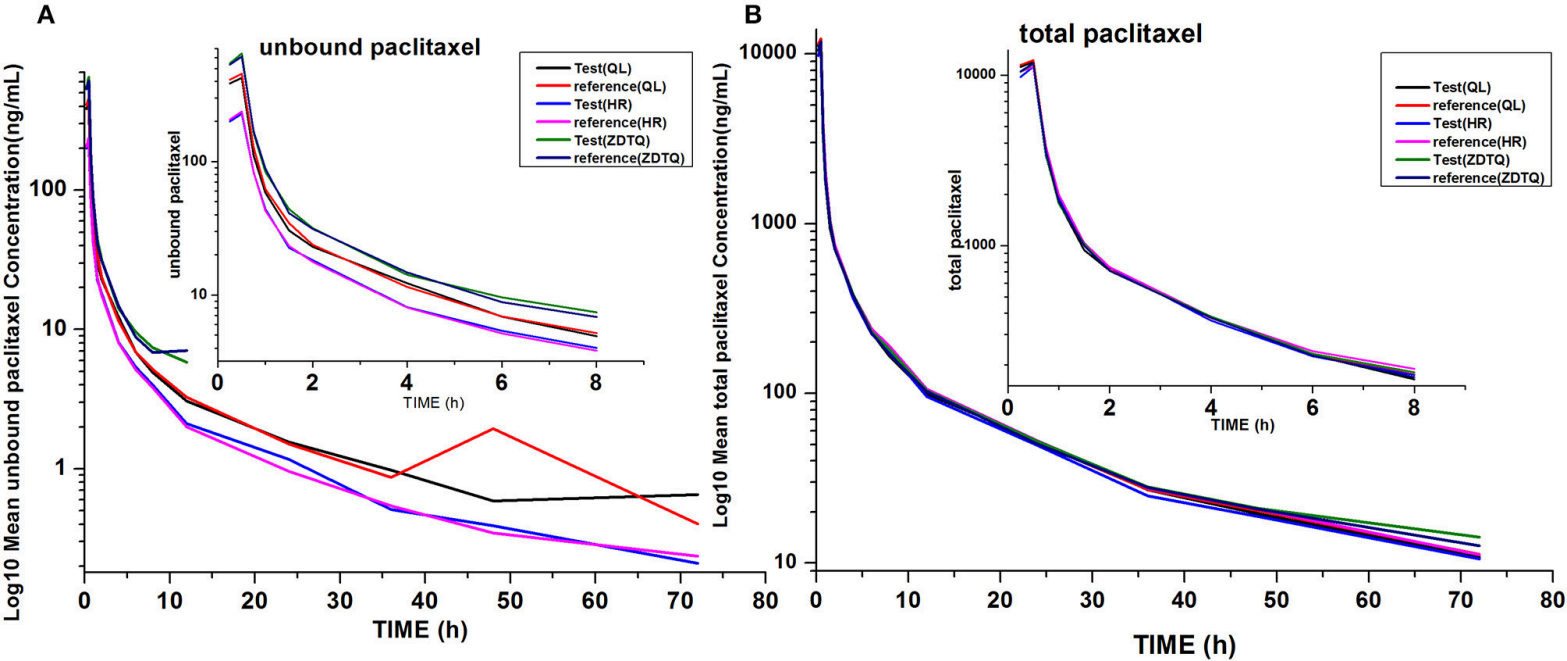
Total LOG10 Mean serum concentration–time profiles in the study. **(A)** total paclitaxel; **(B)** unbound paclitaxel.

AUC_0−t_ accounted for >90% of the AUC_0−∞_ in all subjects, which indicated that the plasma concentration vs. time profiles were well characterized. The mean t_1/2_ were 20.61–27.31 and 20.3–26.74 h, and the intra-cv values ranged from 6.4 to 11% and 9.85 to 15.87% for total paclitaxel and unbound paclitaxel, respectively (except the t_1/2_ of 2.53–3 h of unbound paclitaxel of ZDTQ). The Inter-cv values were small, and almost all of these were less than 30%. There were no differences in PK parameters of total paclitaxel among the Sponsor 1 (QL), Sponsor 2 (HR), and Sponsor 3 (ZDTQ) products, which indicate that food did not affect the PK of paclitaxel. However, the unbound paclitaxel exposure was higher and the elimination rate lower with the Sponsor 1 (QL) and Sponsor 3 (ZDTQ) product than with the Sponsor 2 (HR) product (Table 3, Figure 5). The t_1/2_ of ZDTQ is obviously shorter than those of QL and HR (Table 3, Figure 5).

**Table 3:** Pharmacokinetic parameters of paclitaxel in each study [Geometric Mean (CV%)].

	**Sponsor 1 (QL, light food condition)**		p^*^	**Sponsor2(HR, fasting condition)**		p^*^	**Sponsor3(ZDTQ, light food condition)**		p^*^	**Literature[label]**	***p#***
**PK parameter**	**T (*N* = 25)**	**R (Abraxane®, *N* = 24)**		**T (*N* = 24)**	**R (Abraxane®, *N* = 24)**		**T (*N* = 24)**	**R (Abraxane®, *N* = 24)**		
**UNBOUND PACLITAXEL**											
T_max_ (h)	0.50 (0.25, 0.51)	0.50 (0.25, 0.52)	>0.05	0.5 (0.25, 0.5)	0.5 (0.25, 0.5)	>0.05	0.500 (0.25, 0.50)	0.500 (0.25, 0.50)	>0.05		>0.05
C_max_ (ng/mL)	444.47 (26.37)	476.68 (26.26)	>0.05	234.5 (35.69)	254.5 (36.8)	>0.05	659.95 (17.64)	621.95 (18.30)	>0.05	1284 (41.5)	< 0.05
AUC_0−t_ (h^*^ng/mL)	402.38 (29.11)	440.09 (32.51)	>0.05	252.9 (25.66)	261.8 (25.79)	>0.05	456.54 (21.09)	451.48 (19.54)	>0.05	1159 (29.1)	< 0.05
AUC_0−∞_(h^*^ng/mL)	418.35 (29.62)	456.42 (31.84)	>0.05	259.1 (24.92)	269.2 (25.57)	>0.05	486.66 (20.88)	482.00 (20.47)	>0.05		< 0.05
t_1/2_ (h)	21.60 (43.63)	26.74 (47.09)	>0.05	20.8 (54.27)	20.3 (38.88)	>0.05	2.53 (23.99)	3.00 (69.21)	>0.05		< 0.05
CL/F (L/h)	1105.90 (33.37)	1020.30 (33.82)	>0.05	1670.63 (32.17)	1637.55 (28.14)	>0.05	939.25 (26.13)	953.03 (29.43)	>0.05		< 0.05
Vd/F (L)	34241.78 (50.70)	38522.03 (51.48)	>0.05	50106.05 (66.99)	48635.48 (55.23)	>0.05	3303.71 (19.74)	3791.99 (51.62)	>0.05		< 0.05
**TOTAL PACLITAXEL**											
T_max_ (h)	0.500 (0.25, 0.52)	0.50 (0.25, 0.52)	>0.05	0.5 (0.25, 0.5)	0.5 (0.25, 0.5)	>0.05	0.500 (0.25, 0.50)	0.500 (0.25, 0.50)	>0.05	
C_max_ (ng/mL)	12294.80 (19.28)	12771.25 (16.17)	>0.05	11301 (19.05)	12030 (17.50)	>0.05	11827.50 (15.60)	11905.41 (18.31)	>0.05	19556 (36.2)	>0.05
AUC_0−t_ (h^*^ng/mL)	12196.61 (23.30)	12619.40 (21.83)	>0.05	12025 (19.55)	12730 (16.04)	>0.05	11989.89 (25.41)	11978.56 (25.05)	>0.05	20324 (19.5)	>0.05
AUC_0−∞_ (h^*^ng/mL)	12587.13 (23.29)	13077.56 (22.02)	>0.05	12451 (19.59)	13132 (16.03)	>0.05	12476.88 (24.90)	12405.71 (24.66)	>0.05		>0.05
t_1/2_ (h)	24.48 (18.27)	27.31 (36.96)	>0.05	26.9 (25.20)	24.7 (24.22)	>0.05	21.87 (48.22)	20.61 (34.10)	>0.05	20 (21.3)	>0.05
CL/F (L/h)	35.53 (26.50)	34.09 (24.82)	>0.05	34.39 (25.92)	32.62 (25.17)	>0.05	37.12 (28.28)	37.39 (28.25)	>0.05		>0.05
Vd/F (L)	1253.78 (32.84)	1314.46 (37.19)	>0.05	1304.86 (33.25)	1145.11 (28.43)	>0.05	1131.92 (52.09)	1065.89 (31.90)	>0.05		>0.05

* *paired t test between test (QL and HR) and reference (Abraxane) formation at each sponsor; #, ANOVA test between QL and HR. label: TAXOL® (paclitaxel) Injection*.

### Bioavailability and bioequivalence analysis

The relative bioavailability of the test products as compared with the reference formulation was 92.16–106.44% for unbound paclitaxel and 93.21–100.8% for total paclitaxel. Both the assessments met the 80–125% BE range recommended by the US FDA (Table 4).

**Table 4:** Bioequivalence assessment summary and re-estimation of sample size.

	**Sponsor1 (QL**, ***N*** = **24)**			**Sponsor 2 (HR, *N* = 23)**			**Sponsor 3(ZDTQ**, ***N*** = **24)**	
**PK parameter**	**Intra-CV(%)**	**GMR (90%CI) (%)**	**Re-estimated the sample size**	**Intra-CV(%)**	**GMR (90%CI) (%)**	**Re-estimated the sample size**	**Intra-CV(%)**	**GMR (90%CI)(%)**	**Re-estimated the sample size**
**UNBOUND PACLITAXEL**									
C_max_ (ng/mL)	12.13	93.12 (87.70–98.86)	12	15.87	92.18 (85.04–99.91)	20	9.85	106.44 (101.38–111.75)	9
AUC_0−t_(h^*^ng/mL)	12.2	92.16 (86.77–97.88)	14	12.34	97.05 (91.15–103.32)	10	12.56	101.35 (95.26–107.84)	10
AUC_0−∞_ (h^*^ng/mL)	12.14	92.18 (86.81–97.88)	14	12.38	96.64 (90.75–102.91)	10	12.83	101.41 (95.18–108.04)	10
**TOTAL PACLITAXEL**									
C_max_ (ng/mL)	6.4	94.99 (92.03–98.05)	8	9.76	93.21 (88.70–97.95)	10	6.86	100.02 (96.68–103.48)	6
AUC_0−t_(h^*^ng/mL)	7.81	95.6 (91.98–99.37)	8	10.1	93.26 (88.59–98.17)	10	11	100.29 (94.99–105.90)	9
AUC_0−∞_ (h^*^ng/mL)	8.47	95.28 (91.37–99.36)	8	9.83	93.53 (88.97–98.32)	10	10.96	100.8 (95.49–106.42)	9

* *20% lost to follow-up*.

### Re-estimation of sample size

We re-estimated the sample size for the three studies based on their BE analysis results (α = 0.05, power = 0.8, GMR, and intra-cv) and the original hypothesis. The re-estimated sample size was 6–20, which is less than our enrollment size (Table 4) (Zhang et al., 2018a,b).

### Safety evaluations

For QL product, 25 subjects were included in the safety evaluation. The incidence of AEs was 100% with the QL product and reference product. The incidence of AEs of grade II or higher severity was 32.0% (5/25) vs. 41.6% (10/24) in the QL and reference groups, respectively.

For HR product, 25 subjects were included in the safety evaluation. The incidence of AEs was 100% with the HR product and 91.7% with the reference product. The incidence of AEs of grade II or higher severity was 32.0% (8/25) vs. 37.5% (9/24) in the HR and reference groups, respectively.

For ZDTQ product, 24 subjects were included in the safety evaluation. The incidence of AEs was 100% with the ZDTQ product and the reference product. The incidence of AEs of grade II or higher severity was 83.0% (20/24) vs. 87.5% (21/24) in the HR and reference groups, respectively.

There was one SAE (cataract) with ZDTQ; which was found to be unrelated to the drug. No fatal AEs or study drug injection site reactions of the drugs were observed. The test and reference groups had a similar incidence and pattern of AEs. There were no reports of unexpected AEs. The common hematologic adverse reactions included: neutropenia, leucopenia, thrombocytopenia, and anemia. The common non-hematologic adverse reactions included: increased ALT, AST, and fasting serum glucose levels; hyperesthesia; skin rashes; itching; fever; fatigue; nausea; diarrhea; and vomiting. In these studies, 24 (32.4%) and 23 (31%) subjects had concomitant medication use for co-existing diseases and administration of colony-stimulating factors was the most common group. There was no use of metabolic inducers or inhibitors, such as cyclosporine, phenobarbital, and ketoconazole, among the patients.

## Discussion

In these BE studies, all formulations of albumin-bound paclitaxel (QL, HR, and ZDTQ) were found to be bioequivalent to the reference formulation (Abraxane®) (Slingerland et al., 2013). The most frequently reported AEs were neutropenia, leucopenia, and thrombocytopenia (Slingerland et al., 2013). Following intravenous administration of the study drug (test or reference), paclitaxel serum concentrations declined in a biphasic manner, with the initial rapid decline representing distribution to the peripheral compartment and the slower second phase representing drug elimination^3^ (Slingerland et al., 2013). The terminal half-life of total paclitaxel was about 20.61–27.31 h, which is consistent with the reference product (Abraxane®) label^2^. The large volume of distribution (>1,000 L) of paclitaxel indicates extensive extravascular distribution and/or tissue binding of paclitaxel (Petrelli et al., 2010; Ronghao and Jun, 2015; Hyman et al., 2018; Xiang et al., 2018)^3^. There was higher exposure and lower elimination rate of unbound paclitaxel QL and ZDTQ as compared to HR product.

There are three plausible explanations. First, systematic errors and large standard deviations might have led to this difference. The exposure reported by Sponsor 2 (HR) was lower, regardless of the test and reference formulation. The unbound paclitaxel concentration was obtained with the following formula:

$$\begin{array}{l} {\text{C}}_{\text{unbound paclitaxel}}=\left({\text{C}}_{\text{receiver unbound paclitaxel}}/{\text{C}}_{\text{donor paclitaxel}}\right)\\ \times {\text{C}}_{\text{total paclitaxel}} \end{array}$$

The rapid equilibrium method may have deviation, which might have contributed to the higher unbound paclitaxel concentration when the total paclitaxel concentration was similar. The second reason could be the small difference in binding or wrapping rates. For example, when the binding or wrapping rates of paclitaxel with albumin are 98.0 and 96.5%, which are within the acceptable range, and the total paclitaxel concentration is 12,000 ng/mL; the unbound paclitaxel concentrations will be 240 ng/mL (12,000 × 98.0%) and 420 ng/mL (12,000 × 96.5%), respectively. The third reason could be differences in the metabolism of paclitaxel. Paclitaxel is metabolized into 6α-hydroxypaclitaxel by cytochrome2C8, and to two minor metabolites, 3-*p-*hydroxypaclitaxel, and 6α, 3′-*p*-dihydroxypaclitaxel, by cytochrome 3A4. *In vitro*, the metabolism of paclitaxel to 6α-hydroxypaclitaxel is inhibited by various agents (e.g., verapamil, ketoconazole, vincristine, etc.)^2^; however, none of the patients was taking any such concomitant drug in these studies. Thus, we speculate that a gene polymorphism of CYP could have affected the metabolic capability, which leads to higher clearance and ultimately lower exposure of unbound paclitaxel (Hendrikx et al., 2013; Frederiks et al., 2015; Xie et al., 2015).

As ZDTQ had higher LLOQ, the lower concentration of unbound paclitaxel was not checked 12 h after dosing. The terminal elimination half-life is the time taken by the drug to decrease its plasma concentration to half. The length can reflect the drug elimination rate *in vivo*. If the terminal concentration is very low (the LLOQ is low), the drug elimination terminal slope is small and the half-life is long; if the terminal concentration is high (the LLOQ is high), the drug elimination terminal slope is large and the half-life is short^4^. Then the t_1/2_ of ZDTQ is obviously shorter than those of QL and HR. It can be found at Supplement Figure 1. The two figures of time-concentration PK curve have been drawn from the same subject. We used 0–12 h time-concentration (Supplement Figure 1A) and 0–72 h time-concentration (Supplement Figure 1B) to calculate the t_1/2_. Then we can see the different t_1/2_ (4.7 vs. 21 h).

Food intake did not affect the PK of paclitaxel, which is supported by the similar exposures of total paclitaxel. The US FDA guidance document suggests that if a patient's health status prevents fasting, the clinic trial site may provide a light diet during the study, when all procedures are need to completed under same condition in the bioequivalence study (Davit et al., 2008; Srinivas, 2009).

The intra-subject variability was small as compared with earlier observations among breast cancer patients, i.e., 6.4–15.9 vs. 21.3% for total paclitaxel and unbound paclitaxel, respectively, which further suggests that paclitaxel is a low variable drug (Karalis et al., 2008). Due to the intravenous administration, paclitaxel entered into blood circulation and gastrointestinal absorption was bypassed. The physiological factors can significantly vary not only between subjects but also within the same subject, e.g., luminal/mucosal enzymes, regional pH, biliary or pancreatic secretions, gastric emptying, intestinal motility, and circadian rhythm etc, (Karalis et al., 2008). These factors can contribute to high intra-subject variability of the drug (Shah et al., 1991; Karalis et al., 2008). Then, we re-estimated the sample size of both the studies. We found that these studies did not need a large sample size. In future, we recommend that 22 subjects may be enough, considering the intra-cv measurement (6.4–15.87%) (Shah et al., 1991).

Paclitaxel is known to cause myelosuppression, peripheral neuropathy, myalgia/arthralgia, cardiovascular events, alopecia, and gastrointestinal toxicity (Henderson and Bhatia, 2007; Conlin et al., 2010; Slingerland et al., 2013; Lluch et al., 2014; Li et al., 2015). The severity of neutropenia correlates with the dose of paclitaxel and can be dose-limiting^2^. Dose intensification is not possible in cases of such acute toxicities, and dose reduction may have be necessary to improve a patient's condition, with the possibility of reducing treatment effectiveness. Therefore, albumin-bound paclitaxel was developed with a goal of improving the safety profile of paclitaxel treatment by eliminating the potentially toxic component polyethoxylated castor oil while maintaining or enhancing treatment efficacy (Slingerland et al., 2013). However, if the unbound paclitaxel concentration is associated with these toxicities, it is considered as an indicator to evaluate the technology of the production process (Vishnu and Roy, 2010; Guarneri et al., 2012; Palumbo et al., 2015). When the concentration of unbound paclitaxel is low, it indicates that the paclitaxel is covered by albumin successfully. The incidences of AEs and those of grade II or higher severity were comparable between the test formulation and reference formulation, and both the products were well tolerated by the patients. This shows that the imitation formulation is successful.

## Conclusions

These randomized, two-period, crossover, clinical BE studies show that albumin-bound paclitaxel products (QL, HR, and ZDTQ) are bioequivalent to Abraxane® (reference) with a lower intra-cv and similar safety profiles of among Chinese breast cancer patients.

## Ethics statement

These studies were conducted in accordance with Good Clinical Practice and the Declaration of Helsinki. An independent Ethics Committee (of The First Hospital of Jilin University, Changchun, Jilin, China) approved the protocol before the start of the study. All patients provided informed consent before the start of any study-related procedure.

## Author contributions

XL, QL, HZ, and YD designed the experiment. LG, XL, QL, HZ, MW, XZ and ChL performed the clinic trials. HZ and CuL analyzed the data. QL, HZ and YD wrote and edited the paper, and drew the figures.

### Conflict of interest statement

The authors declare that the research was conducted in the absence of any commercial or financial relationships that could be construed as a potential conflict of interest.

## Footnotes

^1^Available online at: www.rebeccafarm.org

^2^TAXOL® (paclitaxel) INJECTION. https://www.accessdata.fda.gov/drugsatfda_docs/label/2011/020262s049lbl.pdf

^3^Available online at www.accessdata.fda.gov

^4^Pharmacokinetics and Pharmacodynamics Concepts and Applications, Fourth Edition.

## References

[B1] Alves R. C. Fernandes R. P. Eloy J. O. Salgado H. R. N. Chorilli M. (2018). Characteristics, properties and analytical methods of paclitaxel: a review. Crit. Rev. Anal. Chem. 8, 110–118. 10.1080/10408347.2017.1416283 29239659

[B2] Blair H. A. Deeks E. D. (2015). Albumin-bound paclitaxel: a review in non-small cell lung cancer. Drugs 75, 2017–2024. 10.1007/s40265-015-0484-9 26541764

[B3] Conlin A. K. Seidman A. D. Bach A. Lake D. Dickler M. D'Andrea G.. (2010). Phase II trial of weekly nanoparticle albumin-bound paclitaxel with carboplatinand trastuzumab as first-line therapy for women with HER2-overexpressingmetastatic breast cancer. Clin. Breast Cancer 10, 281–287. 10.3816/CBC.2010.n.036 20705560 PMC3883128

[B4] Davit B. M. Conner D. P. Fabian-Fritsch B. Haidar S. H. Jiang X. Patel D. T.. (2008). Highly variable drugs: observations from bioequivalence data submitted to the FDAfor new generic drug applications. AAPS J. 10, 148–156. 10.1208/s12248-008-9015-x 18446515 PMC2751460

[B5] Donehower R. C. Rowinsky E. K. Grochow L. B. Longnecker S. M. Ettinger D. S. (1987). Phase I trial of taxol in patients with advanced cancer. Cancer Treat. Rep. 71, 1171–1177. 2891441

[B6] Dörfel S. Steffens C. C. Meyer D. Tesch H. Kruggel L. Frank M.. (2018). Adjuvant chemotherapeutic treatment of 1650 patients with early breast cancer in routine care in Germany: data from the prospective TMK cohort study. Breast Cancer 25, 275–283. 10.1007/s12282-017-0823-7 29204847 PMC5906523

[B7] Du X. Khan A. R. Fu M. Ji J. Yu A. Zhai G. (2018). Current development in the formulations of non-injection administration ofpaclitaxel. Int. J. Pharm. 542, 242–252. 10.1016/j.ijpharm.2018.03.030 29555439

[B8] FDA (2015). Draft Guidance on Paclitaxel. Available online at https://www.fda.gov/downloads/drugs/guidancecomplianceregulatoryinformation/guidances/ucm320015.pdf

[B9] Frederiks C. N. Lam S. W. Guchelaar H. J. Boven E. (2015). Genetic polymorphisms and paclitaxel- or docetaxel-induced toxicities: asystematic review. Cancer Treat. Rev. 41, 935–950. 10.1016/j.ctrv.2015.10.010 26585358

[B10] Guarneri V. Dieci M. V. Conte P. (2012). Enhancing intracellular taxane delivery: current role and perspectives ofnanoparticle albumin-bound paclitaxel in the treatment of advanced breast cancer. Expert Opin. Pharmacother. 13, 395–406. 10.1517/14656566.2012.651127 22263900

[B11] Henderson I. C. Bhatia V. (2007). Nab-paclitaxel for breast cancer: a new formulation with an improved safetyprofile and greater efficacy. Expert Rev. Anticancer Ther. 7, 919–943. 10.1586/14737140.7.7.919 17627452

[B12] Hendrikx J. J. Lagas J. S. Rosing H. Schellens J. H. Beijnen J. H. Schinkel A. H. (2013). P-glycoprotein and cytochrome P450 3A act together in restricting the oralbioavailability of paclitaxel. Int. J. Cancer 132, 2439–2447. 10.1002/ijc.27912 23090875

[B13] Hyman D. M. Rizvi N. Natale R. Armstrong D. K. Birrer M. Recht L. (2018). Phase I study of MEDI3617, a selective angiopoietin-2 inhibitor alone andcombined with carboplatin/paclitaxel, paclitaxel, or bevacizumab for advancedsolid tumors. Clin Cancer Res. 24, 2749–2757. 10.1158/1078-0432.CCR-17-1775 29559563 PMC6386190

[B14] Joerger M. (2012). Prevention and handling of acute allergic and infusion reactions in oncology. Ann Oncol. 23(Suppl. 10), x313–x319. 10.1093/annonc/mds314 22987983

[B15] Karalis V. Macheras P. Van Peer A. Shah V. P. (2008). Bioavailability and bioequivalence: focus on physiological factors andvariability. Pharm. Res. 25, 1956–1962. 10.1007/s11095-008-9645-9 18551249

[B16] Karalis V. Symillides M. Macheras P. (2012). Bioequivalence of highly variable drugs: a comparison of the newly proposedregulatory approaches by FDA and EMA. Pharm. Res. 29, 1066–1077. 10.1007/s11095-011-0651-y 22203326

[B17] Li Y. Chen N. Palmisano M. Zhou S. (2015). Pharmacologic sensitivity of paclitaxel to its delivery vehicles drives distinct clinical outcomes of paclitaxel formulations. Mol. Pharm. 12, 1308–1317. 10.1021/acs.molpharmaceut.5b00026 25714793

[B18] Lluch A. Alvarez I. Muñoz M. Seguí M. Á. Tusquets I. García-Estévez L. (2014). Treatment innovations for metastatic breast cancer: nanoparticle albumin-bound(NAB) technology targeted to tumors. Crit. Rev. Oncol. Hematol. 89, 62–72. 10.1016/j.critrevonc.2013.08.001 24071503

[B19] Locatelli M. Tinari N. Grassadonia A. Tartaglia A. Macerola D. Piccolantonio S.. (2018). FPSE-HPLC-DAD method for the quantification of anticancer drugs in human wholeblood, plasma, and urine. J. Chromatogr. B Analyt. Technol. Biomed. Life Sci. 1095, 204–213. 10.1016/j.jchromb.2018.07.042 30081349

[B20] Palumbo R. Sottotetti F. Trifirò G. Piazza E. Ferzi A. Gambaro A.. (2015). Nanoparticle albumin-bound paclitaxel (nab-paclitaxel) as second-linechemotherapy in HER2-negative, taxane-pretreated metastatic breast cancerpatients: prospective evaluation of activity, safety, and quality of life. Drug Des. Devel. Ther. 9, 2189–2199. 10.2147/DDDT.S79563 25931813 PMC4404936

[B21] Petrelli F. Borgonovo K. Barni S. (2010). Targeted delivery for breast cancer therapy: the history ofnanoparticle-albumin-bound paclitaxel. Expert Opin. Pharmacother. 11, 1413–1432. 10.1517/14656561003796562 20446855

[B22] Ronghao X. Jun Z. (2015). Chinese Pharmacopoeia. Fourth Edition.

[B23] Shah V. P. Midha K. K. Dighe S. McGilveray I. J. Skelly J. P. Yacobi A.. (1991). Analytical methods validation: bioavailability, bioequivalence andpharmacokinetic studies. Conference report. Eur. J. Drug Metab. Pharmacokinet. 16, 249–255. 10.1007/BF03189968 1823867

[B24] Singla A. K. Garg A. Aggarwal D. (2002). Paclitaxel and its formulations. Int. J. Pharm. 235, 179–192. 10.1016/S0378-5173(01)00986-3 11879753

[B25] Slingerland M. Guchelaar H. J. Rosing H. Scheulen M. E. van Warmerdam L. J. Beijnen J. H.. (2013). Bioequivalence of Liposome-Entrapped Paclitaxel Easy-To-Use (LEP-ETU) formulationand paclitaxel in polyethoxylated castor oil: a randomized, two-period crossover study in patients with advanced cancer. Clin Ther. 35, 1946–1954. 10.1016/j.clinthera.2013.10.009 24290734

[B26] Sparreboom A. van Zuylen L. Brouwer E. Loos W. J. de Bruijn P. Gelderblom H.. (1999). Cremophor EL-mediated alteration of paclitaxel distribution in human blood:clinical pharmacokinetic implications. Cancer Res. 59, 1454–1457. 10197613

[B27] Srinivas N. R. (2009). Considerations for metabolite pharmacokinetic data inbioavailability/bioequivalence assessments. Overview of the recent trends. Arzneimittelforschung 59, 155–165. 10.1055/s-0031-1296380 19517891

[B28] Vishnu P. Roy V. (2010). Nab-paclitaxel: a novel formulation of taxane for treatment of breast cancer. Womens Health 6, 495–506. 10.2217/WHE.10.42 20597612

[B29] Xiang J. Wu B. Zhou Z. Hu S. Piao Y. Zhou Q.. (2018). Synthesis and evaluation of a paclitaxel-binding polymeric micelle for efficient breast cancer therapy. Sci. China Life Sci. 61, 436–447. 10.1007/s11427-017-9274-9 29572777

[B30] Xie J. D. Huang Y. Chen D. T. Pan J. H. Bi B. T. Feng K. Y.. (2015). Fentanyl enhances hepatotoxicity of paclitaxel via inhibition of CYP3A4 and ABCB1 transport activity in mice. PLOS ONE 10:e0143701. 10.1371/journal.pone.0143701 26633878 PMC4669130

[B31] Zhang H. Li Q. Zhu X. Li C. Li X. Liu C.. (2018b). Tolerance, variability, and pharmacokinetics of bevacizumab biosimilars inChinese healthy male subjects. Cancer Chemother. Pharmacol. 82, 615–623. 10.1007/s00280-018-3645-1 30043208

[B32] Zhang H. Li Q. Zhu X. Wu M. Li C. Li X.. (2018a). Association of variability and pharmacogenomics with bioequivalence of gefitinib in healthy male subjects. Front. Pharmacol. 9:849. 10.3389/fphar.2018.00849 30131694 PMC6090208

