# Supplementary material for: Tolerance, Variability and Pharmacokinetics of Albumin-Bound Paclitaxel in Chinese Breast Cancer Patients
Source: Front Pharmacol. 2018 Nov 29;9:1372. doi: 10.3389/fphar.2018.01372 (PMC6284260; doi:10.3389/fphar.2018.01372)
Supplement: Supplementary file 1 [file Table_1.docx]

**Supplement table 1 The quality control in the process of drug concentration detection**

| Sponsor | compound | Concentration range(ng/mL) | RSD(%) | BIAS(%) |
| --- | --- | --- | --- | --- |
| HR | total paclitaxel | 10,900,12000 | 4.2~11.9 | -4.3~1.3 |
|  | unbound paclitaxel | 0.2,5,80 | 4.9~16.1 | -8.4~4.8 |
| QL | total paclitaxel | 15,900,12000 | 4.9~5.7 | -28.7~6.0 |
|  | unbound paclitaxel | 0.6,8,160 | 5.1~5.9 | -23.5～1.5 |
| ZDTQ | total paclitaxel | 30,350,4000,8000 | 4.8~6.5 | -2.9~-1.6 |
|  | unbound paclitaxel | 6,100,750,1500 | 6.6~7.5 | -2.8~2.5 |

BIAS(%)=(measured value - true value)/ true value *100%

RSD(%)=The standard deviation of measured value/the MEAN of measured value*100%


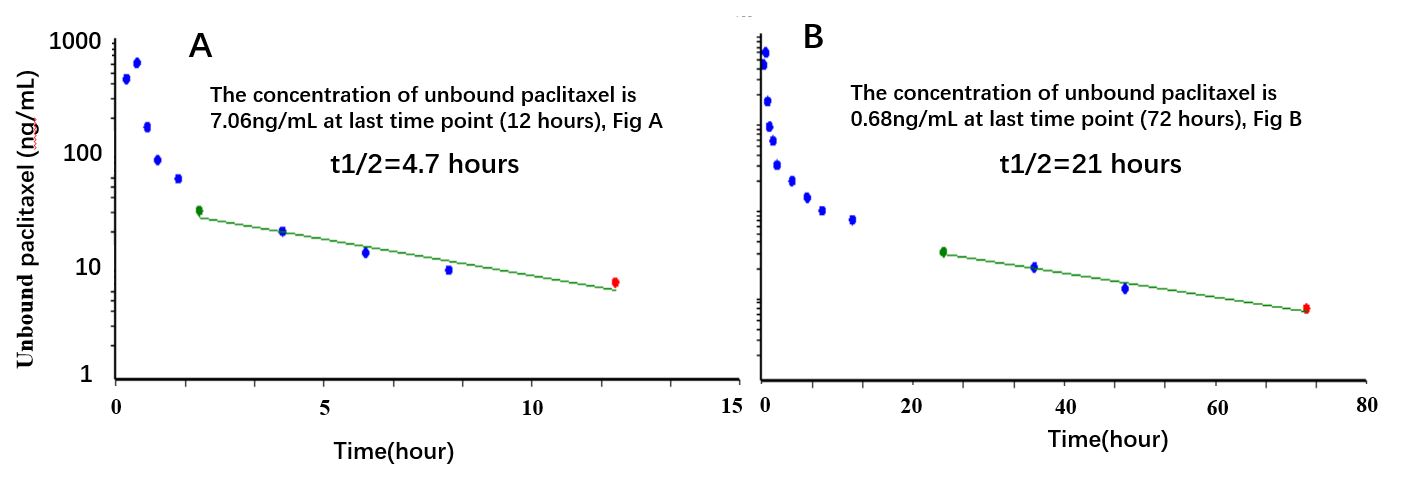


**Supplement Fig. 1 The concentration–time profiles of unbound paclitaxel of one subject.** 0-12 hour time-concentration (Supplement Fig.1 A); 0-72 hour time-concentration (Supplement Fig.1 B);
